# MISSING DEMENTIA DIAGNOSIS INFORMATION DURING HOME HEALTH: PREVALENCE AND IMPACTS

**DOI:** 10.1093/geroni/igae098.2158

**Published:** 2024-12-31

**Authors:** Julia Burgdorf

**Affiliations:** VNS Health, New York, New York, United States

## Abstract

Medicare-funded Home Health Care (HHC) delivers skilled health care services through visits to patients’ homes. Poor information transfer between HHC and other care settings is a perennial issue; HHC providers rarely have reliable and complete information regarding patients’ clinical history or needs. One-third of HHC patients have dementia, but lack of visibility into a patient’s dementia status could negatively impact HHC providers’ ability to appropriately tailor care to these individuals. We examined a national sample of 426,606 older adults (65+) who received Medicare HHC in 2018 and had diagnosed dementia, as reported in the Medicare Beneficiary Summary File. The majority (68.3%) did not have their dementia diagnosis captured in the HHC claims or assessment files. We fit multilevel logistic models clustering at the HH agency-level and adjusting for patient sociodemographic characteristics and clinical status and HHC agency characteristics. Patients whose dementia diagnosis was not captured during HHC were less likely to receive Speech-Language Pathology (aOR: 0.63; 95% CI: 0.61-0.64), social work (aOR: 0.70; 0.69-.72), or aide visits (aOR: 0.81, 95% CI: 0.80-0.82). Patients whose dementia diagnosis was not captured were also more likely to be hospitalized (aOR: 1.69; 1.65-1.73) or to visit the Emergency Department (aOR: 1.47; 95% CI: 1.44-1.50) during HHC, and less likely to have their Activities of Daily Living (ADL) limitations stabilize or improve by HHC discharge (aOR: 0.70; 95% CI: 0.68-0.72). Results suggest HHC providers often lack pertinent information regarding patients’ dementia status, contributing to reduced access to relevant services and increased risk for negative outcomes.

